# Optimization of cone beam computed tomography image quality in implant dentistry

**DOI:** 10.1002/cre2.141

**Published:** 2018-11-12

**Authors:** Yasmine Alawaji, David S. MacDonald, Georgios Giannelis, Nancy L. Ford

**Affiliations:** ^1^ Department of Oral Biological and Medical Sciences, Faculty of Dentistry The University of British Columbia British Columbia Canada; ^2^ Department of Physics and Astronomy, Faculty of Science The University of British Columbia British Columbia Canada

**Keywords:** clinical image evaluation, dental CBCT, image quality, implant treatment planning, radiation dose

## Abstract

This study was conducted to optimize the cone beam computed tomography image quality in implant dentistry using both clinical and quantitative image quality evaluation with measurement of the radiation dose. A natural bone human skull phantom and an image quality phantom were used to evaluate the images produced after changing the exposure parameters (kVp and mA). A 10 × 5 cm^2^ field of view was selected for average adult. Five scans were taken with varying kVp (70–90 kVp) first at fixed 4 mA. After assessment of the scans and selecting the best kVp, nine scans were taken with 2–12 mA, and the kVp was fixed at the optimal value. A clinical assessment of the implant‐related anatomical landmarks was done in random order by two blinded examiners. Quantitative image quality was assessed for noise/uniformity, artifact added value, contrast‐to‐noise ratio, spatial resolution, and geometrical distortion. A dosimetry index phantom and thimble ion chamber were used to measure the absorbed dose for each scan setting. The anatomical landmarks of the maxilla had good image quality at all kVp settings. To produce good quality images, the mandibular landmarks demanded higher exposure parameters than the maxillary landmarks. The quantitative image quality values were acceptable at all selected exposure settings. Changing the exposure parameters does not necessarily produce higher image quality outcomes but does affect the radiation dose to the patient. The image quality could be optimized for implant treatment planning at lower exposure settings and dose than the default settings.

## 1 INTRODUCTION

Indications for cross‐sectional x‐ray imaging modalities in implant treatment planning have been controversial, with many organizations, such as the European Association for Osseointegration, believing that conventional radiographic modalities are adequate for implant treatment planning and the increased dose that results from cone beam computed tomography (CBCT) is not justified (Harris et al., 2012). On the other hand, Tyndall et al. (2012) recommend CBCT use in three phases of implant therapy: during initial assessment, preoperative evaluation, and postoperative evaluation. The use of CBCT in implant treatment planning became more common after the publication of reports of serious or life‐threatening complications due to invasion of anatomical landmarks (Jacobs, Quirynen, & Bornstein, 2014; Renton, Dawood, Shah, Searson, & Yilmaz, 2012; Zijderveld, van den Bergh, Schulten, & ten Bruggenkate, 2008).

In light of the concerns about radiation dose from CBCT imaging, dose optimization studies have been reported, with a couple of recent review articles summarizing previous studies nicely (Goulston, Davies, Horner, & Murphy, 2016; McGuigan, Duncan, & Horner, 2018). In a systematic review, the need for consistent test objects, methodologies, and reporting of techniques was identified for better comparison between studies; despite these shortcomings, the authors concluded that CBCT dose reduction should be possible without compromising the diagnostic quality, at least for some imaging tasks (Goulston et al., 2016). McGuigan et al. (2018) concluded that optimization of kV and mA is challenging to balance the image quality with radiation dose and noted a need to relate the objective metrics of image quality to clinical tasks and radiation dose. The radiation dose should be maintained as low as reasonably achievable (ALARA; ICRP, 2007)—not so low to negatively affect the diagnostic value or higher than required, leading to excessive unjustified radiation.

Optimization of CBCT image quality was done using different approaches in the literature. Some studies used dry human skulls or patient scans to optimize clinical image quality. The CBCT scans were taken at variable exposure parameters, and the images were evaluated for diagnostic values (Dawood, Brown, Sauret‐Jackson, & Purkayastha, 2012; Lofthag‐Hansen, Thilander‐Klang, & Grondahl, 2011; Shelley, Brunton, & Horner, 2011). This clinical assessment was solely subjective and lacks the control of corresponding radiation exposure.

A quality assurance program, the SEDENTEX Project, was developed by the European Union. It requires the use of an image quality phantom to assess several physical properties such as contrast‐to‐noise ratio (CNR), geometrical distortion, spatial resolution, noise, uniformity, and artifact added value (AAV). Quantitative investigations of image quality have been reported, using SEDENTEXCT image quality (IQ) phantom as well as SEDENTEXCT dosimetry index (DI) phantom at variable exposure protocols (Abouei, Lee, & Ford, 2015; Ford, Sonya, & Davies, 2016; Pauwels et al., 2011; Sedentex Project, 2012). Pauwels et al. (2014) and Pauwels et al. (2015) concluded that dose reduction can be attained without affecting the image quality by reducing the exposure settings.

The translation of the clinical application of the quantitative image quality, however, is also subjective in nature. Few studies attempted to study both quantitative and clinical image quality assessments and correlate them (Choi et al., 2015; Dawood et al., 2012; Lofthag‐Hansen et al., 2011; Pauwels et al., 2015; Shelley et al., 2011). None of these studies measured the corresponding dose to optimize the image quality, and they attempted to assess several dental tasks together to come to an overall conclusion. In a recent study, Al‐Okshi, Lindh, Sale, Gunnarsson, and Rohlin (2015) describe their method of optimization for assessment of periodontal structures in CBCT. This study used measured CNR, dose‐area product and performed an observer study to assess periodontal bone quality at three sites for each of 14 teeth. From their study, technique factors including 80 kVp, 5 mA, and 17.5‐s exposure time were suggested as optimal for their periodontal imaging task.

The aim of this study is to optimize the image quality of CBCT in implant dentistry comprehensively using clinical and quantitative image quality assessments to achieve the best images at the lowest possible dose. We will measure the absorbed dose and the complete set of image quality metrics (noise, uniformity, CNR, spatial resolution, artifacts, and geometric distortion) as outlined by the Sedentex Project (2012). We will also perform an observer study to assess the diagnostic value of the images for tasks related to implant dentistry. The outcome of the study will be a recommendation for optimized technique settings specific to implant dentistry.

## 2 MATERIALS AND METHODS

### 2.1 CBCT machine

A dental CBCT scanner (CS 9300, Carestream Health, Inc., Rochester, NY, USA) at the Faculty of Dentistry, University of British Columbia, was used for the scans. A 10 × 5 cm^2^ field of view (FOV) was selected in this study as it is a commonly used FOV in implant planning. The default setting for this FOV for an average adult patient is 90 kVp, 4.0 mA, 6.20 s, and a voxel size of 180 μm.

### 2.2 Data acquisition protocol

**Figure 1:**
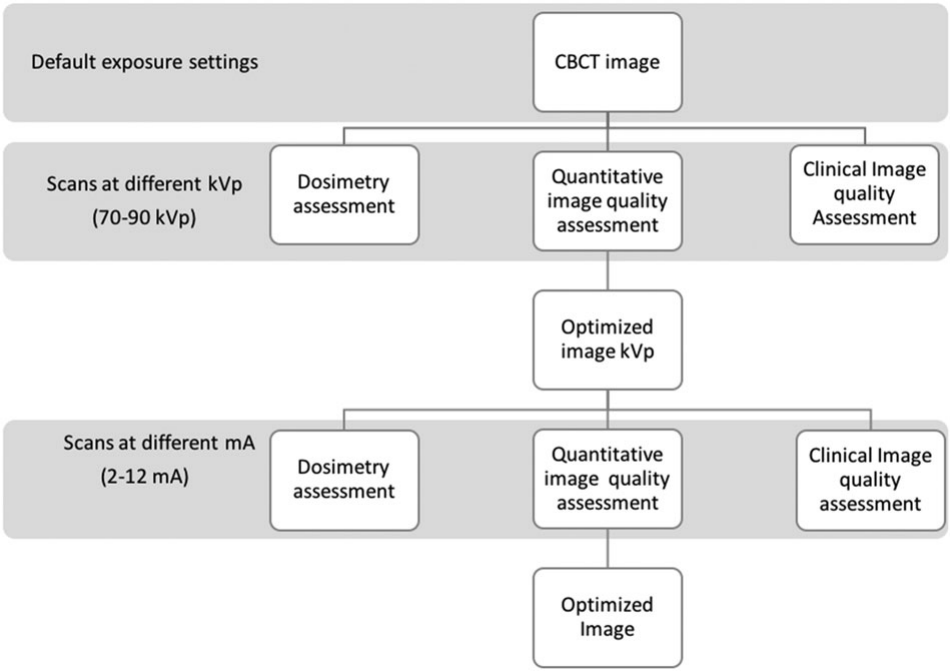
Flow chart illustrates the sequence of assessment and optimization of the image quality. CBCT: cone beam computed tomography

Three types of phantoms were used in this study for three types of assessments: clinical image quality assessment, physical image quality assessment, and dosimetry. The CBCT scans were taken at five different tube potentials: 90, 85, 80, 75, and 70 kVp. Other parameters were fixed at 4.0 mA, 6.20 s, and a voxel size of 180 μm. The scans were taken using each phantom separately following the same protocol of exposure settings. A comprehensive analysis of dosimetry, quantitative image quality, and clinical image quality was done to select scans with good image quality at the lowest possible kVp and dose. After selection of the optimal kVp, scans were taken at nine different tube currents, 2, 2.5, 3.2, 4, 6.3, 8, 10, and 12 mA at fixed kVp, voxel size, and time. All scans were evaluated for the dosimetry, quantitative image quality, and clinical image quality to select the good quality images at the lowest possible mA and dose. The sequence of the protocol to optimize the image quality is illustrated in Figure 1.

### 2.3 Dosimetry

**Figure 2:**
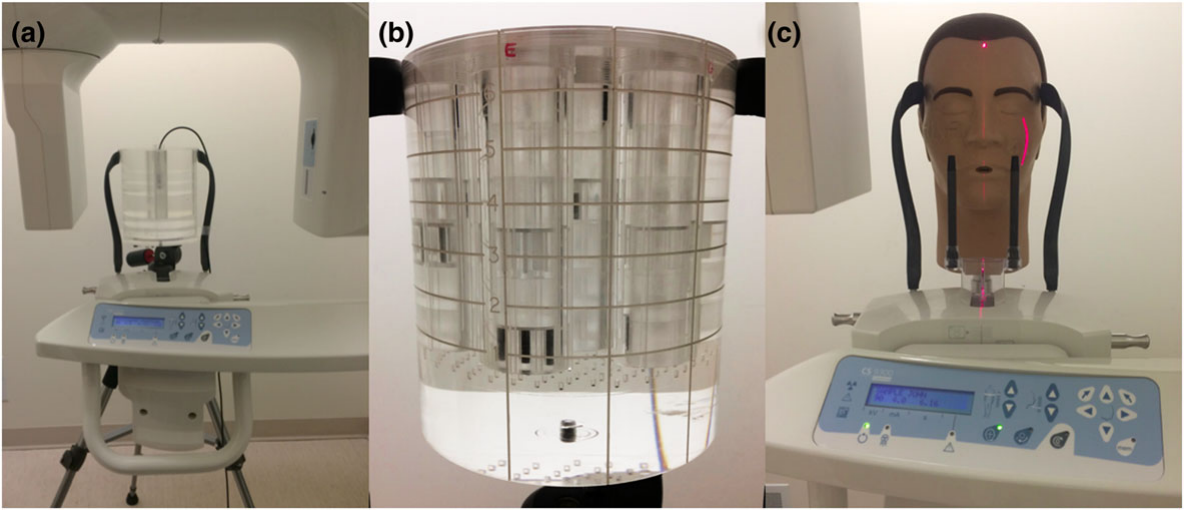
(a) Dosimetry index phantom and thimble ionization chamber, (b) image quality phantom, and (c) PAN DXTTR positioned in the field of view to scan the maxillary arch

The absorbed dose was measured at the five different kVp settings and nine different mA settings with fixed exposure time of 6.20 s and voxel size of 180 μm, as described above. The dose index phantom was used (SEDENTEXCT DI, Leeds Test Objects Ltd., Boroughbridge, UK; Figure 2a). The phantom is composed of six stacks of polymethyl methacrylate (PMMA) plates that simulate human tissue density (1.20 ± 0.01 g/cm^3^). The cylinder size was of head size (160‐mm diameter and 176‐mm height). The dose measurement method was done according to the SEDENTEXCT project (Sedentex Project, 2012). Five different regions along the phantom diameter were selected to measure the dose. The FOV was at the level of the center slice of the DI phantom. A calibrated 0.6‐cm^3^ thimble ionization chamber (10 × 6–0.6 CT, Radcal Corporation, Monrovia, USA) was placed into a hollow column at each of selected five regions at the level of the center slice of the phantom. Two measurements of the dose using the thimble ionization chamber were taken at five different regions along gradient of dose profile. The average of measurements was used to calculate the absorbed dose.

### 2.4 Quantitative image quality

The image quality phantom, shown in Figure 2b, was manufactured by Leeds Test Objects Ltd. (SEDENTEXCT IQ), and the phantom and associated image quality tests have been described previously (Abouei et al., 2015; Bamba, Araki, Endo, & Okano, 2013; Ford et al., 2016; Pauwels et al., 2011). Five essential physical image quality parameters were used in the assessment of physical image quality of each scan, including noise/uniformity, CNR, AAV, geometrical distortion, and spatial resolution. The analysis was done using ImageJ software (NIH Inc, Bethesda, Maryland, USA). The noise is taken as the mean of the standard deviation measured in five circular regions of interest (ROIs) in the PMMA cylinder. The uniformity was measured by subtracting the mean gray value (MGV) of four peripheral ROIs from the central circular ROI. The contrast was measured for each material as compared with the PMMA background for aluminum, polytetrafluoroethylene, polyoxymethylene, low‐density polyethylene, and air. The CNR is the contrast divided by the average noise in the image. Two line profiles perpendicular to each other were plotted in the geometric distortion layer, and the distance between voids was measured using MATLAB software (Mathworks, Natick, Massachusetts, USA). Any deviation in the measurements between the voids from the actual distance (10 mm) is considered geometrical distortion. To measure the AAV, the MGV in two rectangular ROIs adjacent to the rods was measured compared with the MGV of background. The spatial resolution was measured using both quantitative and qualitative methods. The qualitative method was done by visual inspection of two polymer line chart inserts located along the *Z*‐direction and in the *XY*‐plane. The spatial resolution was calculated quantitatively using the point spread function following the method described by Abouei et al. (2015). A square ROI was placed around the wire, and the resulting distribution was plotted using MATLAB software. The full width at half maximum was calculated. The point spread function was used to calculate modulation transfer function (MTF) using the fast Fourier transform. The spatial resolution was calculated using the frequency at 10% of MTF.

### 2.5 Subjective image quality

A PAN DXTTR® natural manikin (Dentsply Rinn, York, Pennsylvania, USA) with a dry human skull embedded into resin was used (Figure 2c). The phantom is mounted on a metal tripod with wheel base adjustable up to 5′6″ height. The extraoral landmarks of the phantom are accurate to position the skull using the Frankfort and midsagittal planes. The clinical image quality assessment was done through evaluation of selected essential anatomical landmarks that involves vital structures in both maxilla and mandible. Nine maxillary and 10 mandibular structures were selected based on the anatomy of the skull phantom and are listed in Table 1. The clinical image quality assessment included relevant tasks, such as tracing the mandibular canal and measurements of bone thickness at different locations, and some additional landmarks to assess the image quality across the entire image volume.

**Table 1:** Selected anatomical landmarks commonly used for presurgical implant planning

Maxillary anatomical landmarks	Mandibular anatomical landmarks
Nasal spine	Interalveolar lateral foramen (right side)
Incisive canal	Interalveolar medial foramen (left side)
Buccal plate thickness at tooth #21 at the crest	Inferior lingual foramen
Buccal plate thickness at tooth #22 at midpoint apico‐coronally	Superior lingual foramen
Buccal plate thickness at tooth #23 apically	Interproximal bone height between teeth #33 and #32
Interproximal bone height between teeth #13 and #14	Buccal plate thickness of tooth #41 apically
Antroalveolar anastomosis at lateral maxillary sinus wall (left side)	Buccal plate thickness of tooth #42 midpoint apico‐coronally
Antroalveolar anastomosis at lateral maxillary sinus wall (right side)	Buccal plate thickness of tooth #43 crestally
	Mental foramen left side
Sinus septum	Tracing of the mandibular canal (right)

Five images were taken of each arch at five different kVps (70, 75, 80, 85, and 90 kVp). Two examiners evaluated the clinical image quality in a random order using a 5‐point Likert scale (excellent, good, adequate, poor, and undetectable; Likert, 1932). The examiners who performed the assessment were blinded to the parameters of the scans and are an experienced oral and maxillofacial radiologist and an experienced periodontist. Randomized order of the images was generated using MATLAB software, version 2014 (Mathworks Inc.). Excellent, good, and adequate scores of detecting and tracing the anatomical landmark in three‐dimensional assessment were considered as high image quality. Each examiner did the examination separately using the same monitor and under the same lighting conditions. The assessment of different kVp was done twice by each examiner, and they were given the choice of changing the brightness and contrast of the images.

The images at different kVp were evaluated for the best clinical and quantitative image quality at lowest possible dose. Then, nine images of each arch were taken at nine different mA settings: 2, 2.5, 3.2, 4, 5, 6.3, 8, 10, and 12 mA. The images were assessed for the clinical and quantitative image quality following the same protocol used to assess images at different kVp.

## 3 RESULTS

### 3.1 Dose measurement

**Figure 3:**
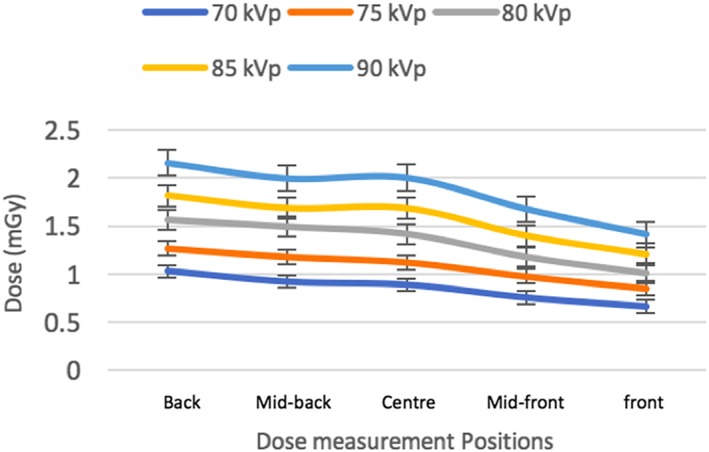
Dose measurements at different kVp

**Figure 4:**
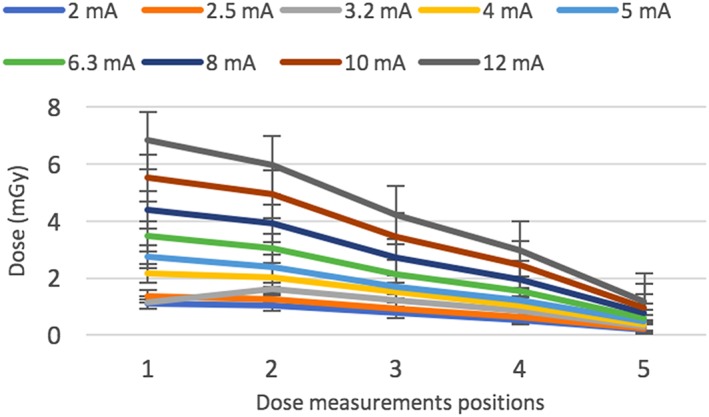
Dose measurements at different mA

The radiation absorbed dose increased with increase in peak kilovoltage and tube current settings. The dose measurements at the five selected regions along the phantom profile at all exposure settings are represented in Figures 3 and 4. The exposure settings of choice were selected based on the lowest possible dose that have good clinical as well as quantitative image quality. The default settings of 90 kVp and 4 mA had a dose equal to 1.9 ± 0.3 mGy.

### 3.2 Quantitative image quality assessment

**Figure 5:**
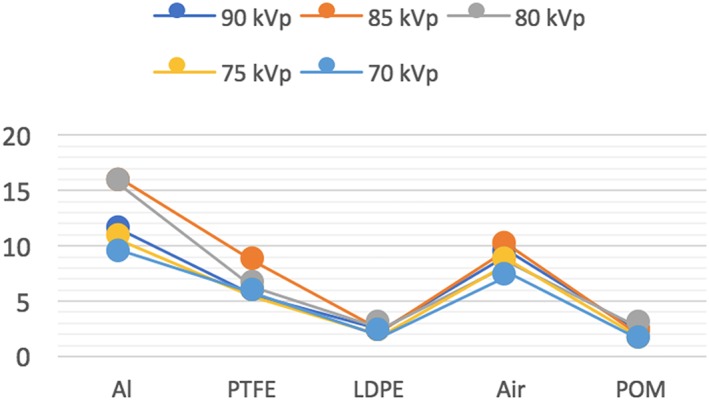
Contrast‐to‐noise ratio at different kVp. Al: aluminum; PTFE: polytetrafluoroethylene; LDPE: low‐density polyethylene; POM: polyoxymethylene

**Figure 6:**
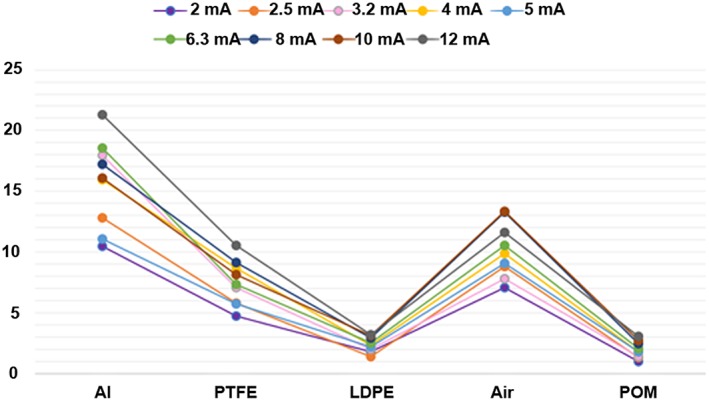
Contrast‐to‐noise ratio at different mA. Al: aluminum; PTFE: polytetrafluoroethylene; LDPE: low‐density polyethylene; POM: polyoxymethylene

**Figure 7:**
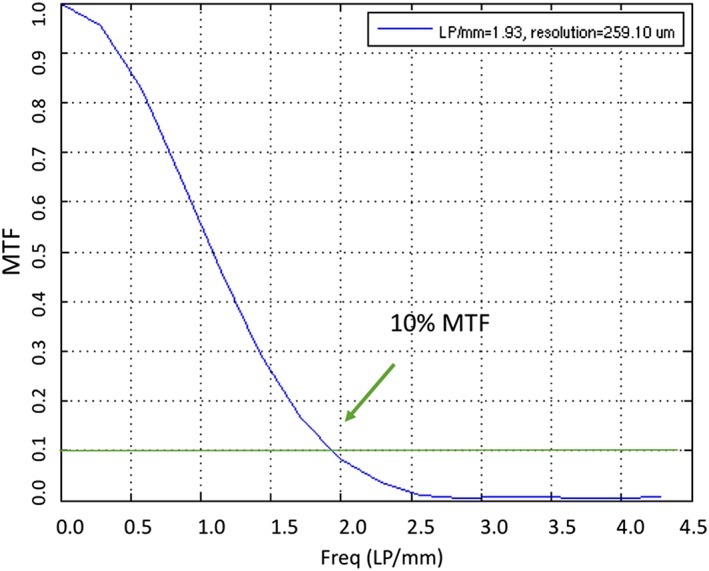
Resolution (0.26 mm) at 10% modulation transfer function (MTF)

The noise as measured by the standard deviation from the MGV increased with decrease in both kVp and mA. The uniformity had the best value at 80 kVp among different kVp settings. When uniformity was measured at different mA, it had the best value at 4 mA. AAV had the best result with lowest value at 90 kVp. After assessment of AAV at different mA settings, the best value was found at 4 mA. CNR had the best values at 85 kVp for aluminum, polytetrafluoroethylene, and air. The 80 kVp had the best CNR values for materials with low contrast, the low‐density polyethylene and polyoxymethylene. CNR had the best value at 12 mA for all materials except air as it had the best value at 8 mA (Figures 5 and 6). Spatial resolution showed small improvements with increased kV and mA settings for MTF (Figure 7) and visual line pair assessment, whereas the geometrical distortion did not differ with variable exposure settings. Quantitative image quality values of different kVp settings are listed in Table 2 and at different mA settings in Table 3.

**Table 2:** Quantitative image quality values at different kVp

kVp		90	85	80	75	70
Noise (*SD*)		88.1	97.1	115.3	131.9	152.7
Uniformity		81.5	84.9	80.7	82.9	85.6
Geometrical distortion	1st line ± *SD*	10.0 ± 0.5	9.9 ± 0.4	10.0 ± 0.3	10.0 ± 0.3	10.0 ± 0.3
	2nd line ± *SD*	10.0 ± 0.4	10.0 ± 0.4	10.0 ± 0.2	10.0 ± 0.5	10.1 ± 0.3
Artifact 1		331.0	397.9	370.6	452.9	457.8
Artifact 2		164.2	168.1	192.7	227.3	282.4
Limiting resolution from MTF (lp/mm)		1.9	1.9	1.8	1.9	1.7
Effective voxel size (mm)		0.27	0.27	0.28	0.27	0.29
Resolution from *XY* line pattern (lp/mm)		1.0	1.0	1.0	1.0	1.0
Resolution along *Z*‐axis from line pattern (lp/mm)		1.7	1.7	1.0	1.0	1.0

*Note*. MTF: modulation transfer function.

**Table 3:** Quantitative image quality values at different mA

mA		2	2.5	3.2	4	5	6.3	8	10	12
Noise (*SD*)		157.5	140.3	121.7	97.1	98.4	95.1	79.6	70.7	65.8
Uniformity		−35.9	−10.6	26.8	44.0	79.5	52.3	34.9	−18.4	−27.5
Geometrical distortion	1st line ± *SD*	10.0 ± 0.3	10.1 ± 0.3	10.1 ± 0.4	10.0 ± 0.3	10.1 ± 0.4	10.1 ± 0.2	10.1 ± 0.3	10.0 ± 0.4	10.0 ± 0.1
	2nd line ± *SD*	9.9 ± 0.5	10.0 ± 0.4	10.0 ± 0.4	10.0 ± 0.5	10.0 ± 0.4	10.0 ± 0.5	10.0 ± 0.3	10.0 ± 0.4	10.0 ± 0.3
Artifact 1		243.6	379.1	417.8	397.9	521.9	412.2	441.0	356.3	422.0
Artifact 2		292.5	249.6	227.4	298.8	282.7	265.2	276.4	297.1	303.0
Limiting resolution from MTF (lp/mm)		1.9	1.9	1.9	1.9	1.9	1.9	1.8	1.8	1.7
Effective voxel size (mm)		0.27	0.27	0.27	0.27	0.27	0.27	0.28	0.27	0.29
Resolution from *XY* line pattern (lp/mm)		1.0	1.0	1.0	1.0	1.0	1.0	1.0	1.7	1.7
Resolution along *Z*‐axis from line pattern (lp/mm)		1.0	1.7	1.7	1.7	1.7	1.7	1.7	1.7	1.7

*Note*. MTF: modulation transfer function.

### 3.3 Subjective image quality assessment

**Figure 8:**
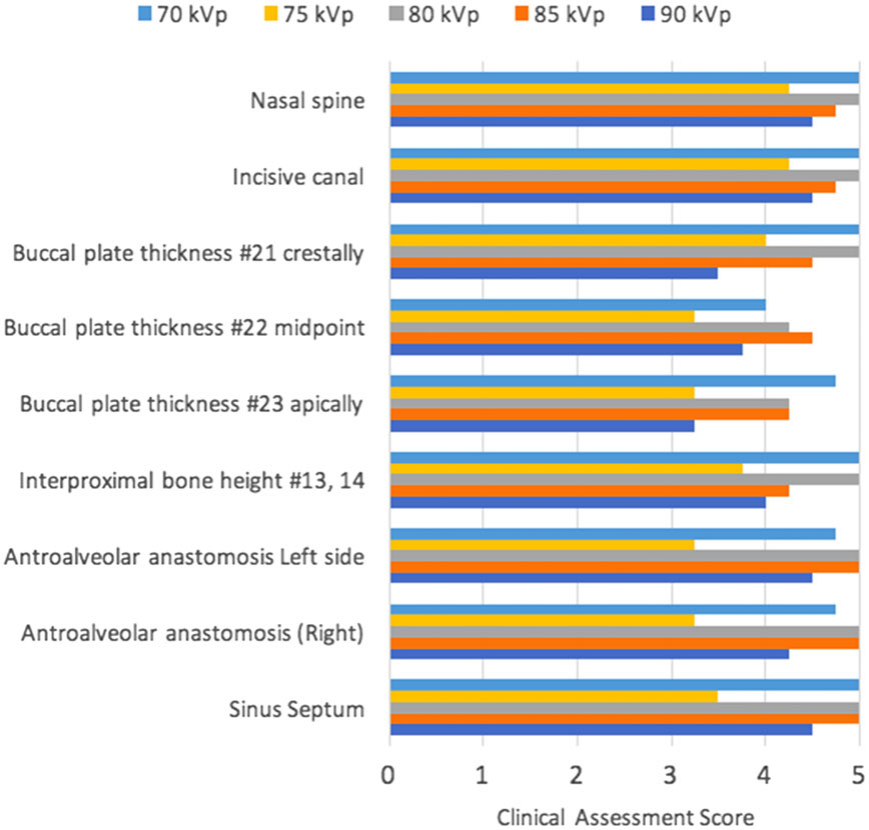
Maxillary landmark assessment at different kVp

**Figure 9:**
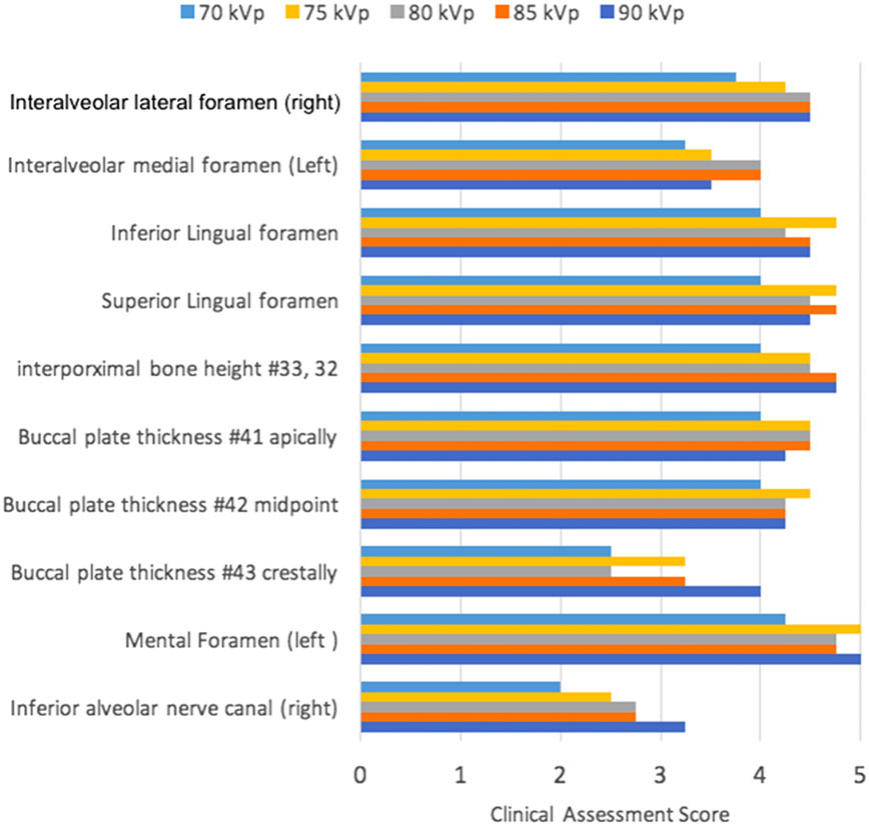
Mandibular landmark assessment at different kVp

**Figure 10:**
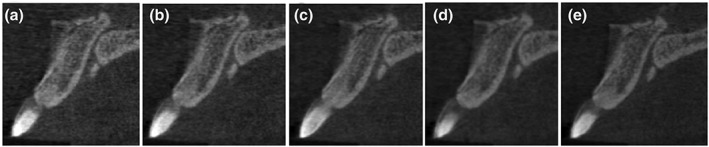
Nasopalatine canal and nasal spine at different kVp settings: (a) 70 kVp, (b) 75 kVp, (c) 80 kVp, (d) 85 kVp, and (e) 90 kVp

**Figure 11:**
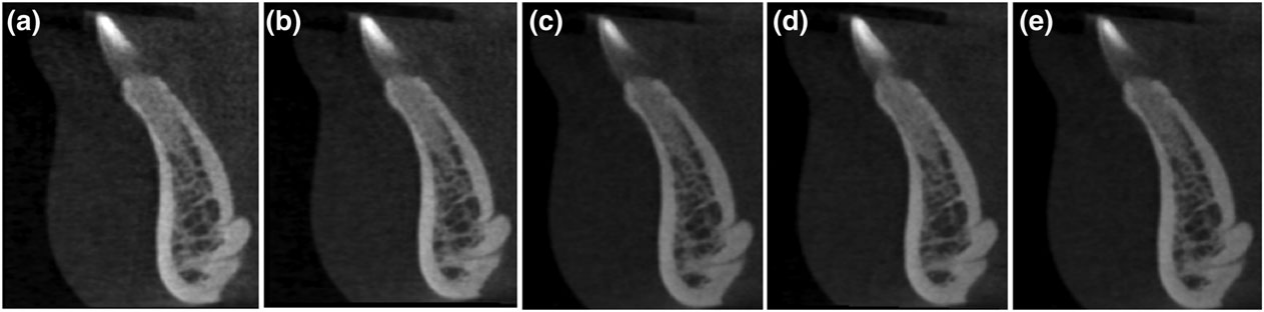
Superior and inferior lingual foramina at different kVp settings: (a) 70 kVp, (b) 75 kVp, (c) 80 kVp, (d) 85 kVp, and (e) 90 kVp

**Figure 12:**
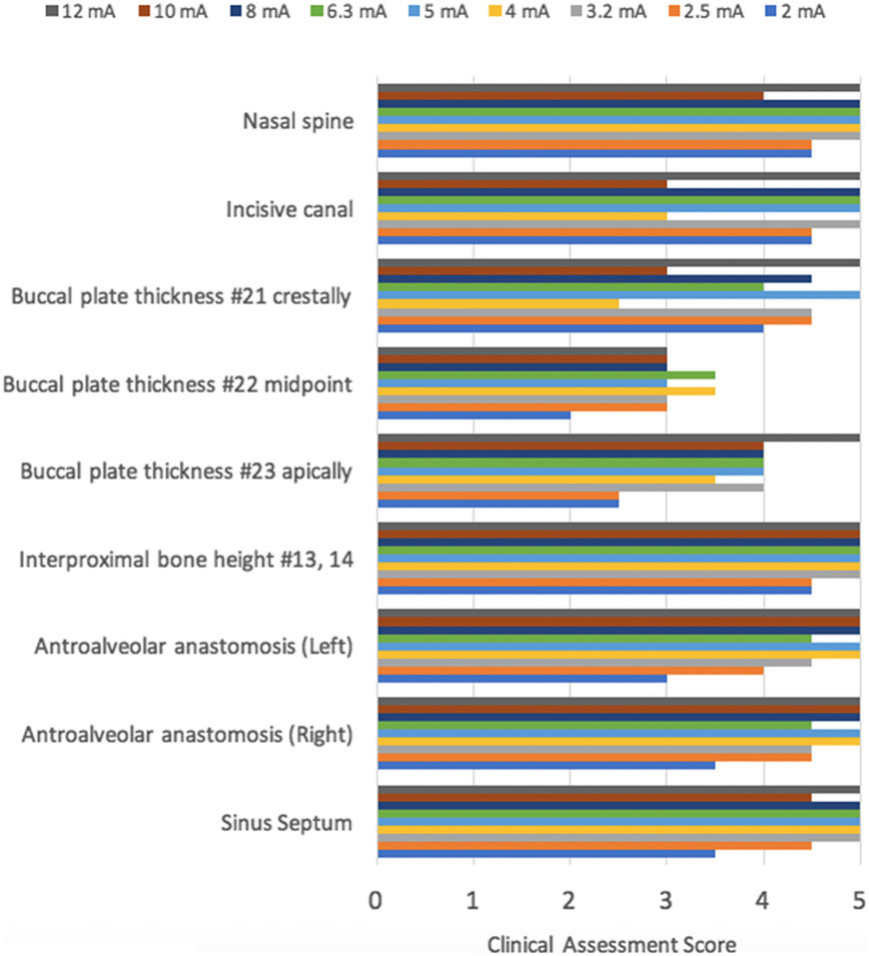
Maxillary landmark assessment at different mA

**Figure 13:**
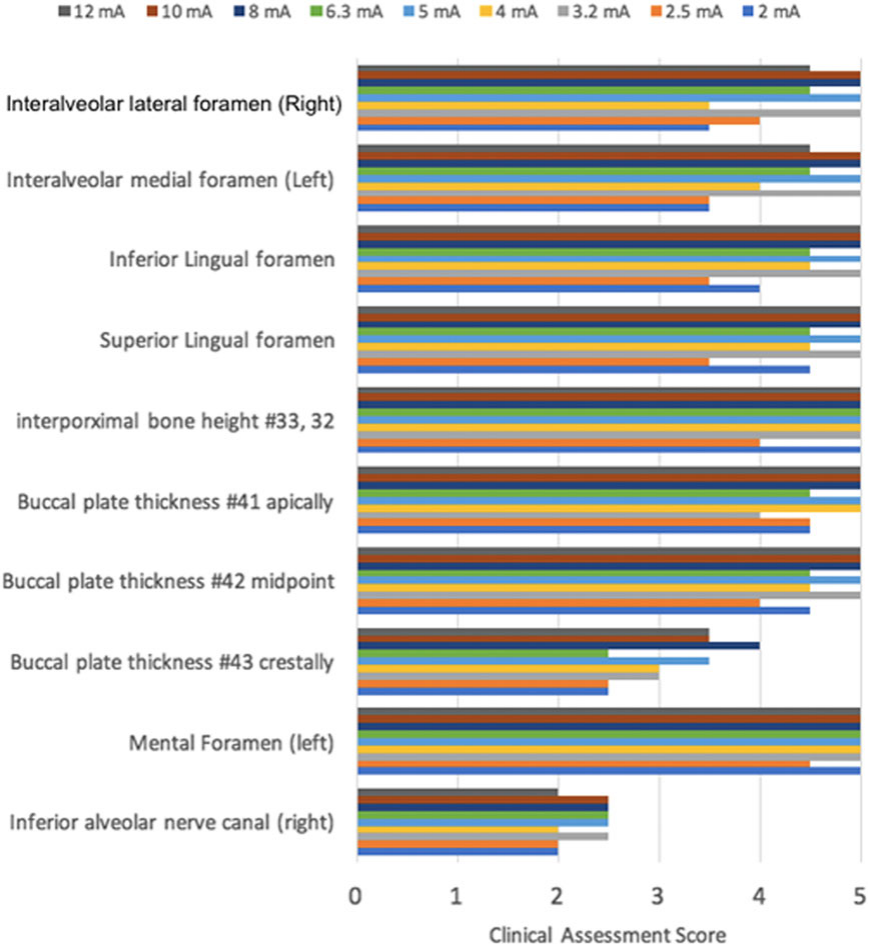
Mandibular landmark assessment at different mA

**Figure 14:**
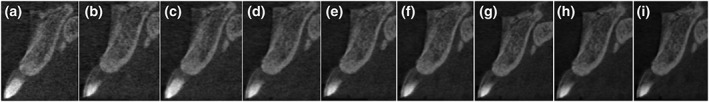
Nasopalatine and nasal spine at different mA settings: (a) 2 mA, (b) 2.5 mA, (c) 3.2 mA, (d) 4 mA, (e) 5 mA, (f) 6.3 mA, (g) 8 mA, (h) 10 mA, and (i) 12 mA

**Figure 15:**
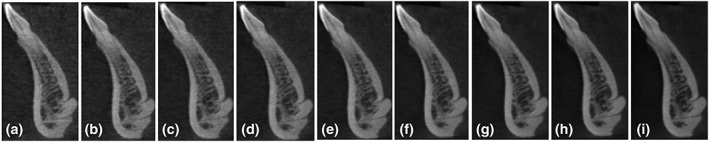
Superior and inferior lingual foramina: (a) 2 mA, (b) 2,5 mA, (c) 3.2 mA, (d) 4 mA, (e) 5 mA, (f) 6.3 mA, (g) 8 mA, (h) 10 mA, and (i) 12 mA

The landmarks that had a score of 3 or more were considered to have good image quality. The viewers evaluated the anatomical structures for the clarity of identification as well as tracing the structures in all three‐dimensional cross sections. The evaluated maxillary landmarks had good quality at all different kVp settings from 90 to 70 kVp. The overall subjective evaluation of mandibular landmarks had good quality at 85 and 90 kVp. The inferior alveolar canal was clearly seen at three different cross sections. However, the tracing of the landmark continuously in any plane was impossible at all settings. The 85 kVp was selected as the optimal kVp as it is the lowest possible kVp that worked for the maxillary and mandibular landmarks. The clinical assessment of both maxillary and mandibular landmarks at different kVp is shown in Figures 8 and 9. Sample images are shown at different kVp settings (Figures 10 and 11). After changing the mA at fixed 85‐kVp setting, the clinical assessment of maxillary landmarks had good quality at different mA from 3.2 mA and above. The inferior alveolar canal was still not possible to trace continuously for all different mA settings. The buccal thickness of lower right canine had poor visibility at 6.3, 2.5, and 2 mA. The clinical assessment of maxillary and mandibular landmarks at mA is illustrated in Figures 12 and 13. A sample of clinical images at different mA settings is shown (Figures 14 and 15). The optimal mA setting was 3.2 mA where all maxillary and mandibular landmarks had good quality.

### 3.4 Optimization

For kVp optimization, the image quality metrics showed more improvements at higher kVp (reduced noise and artifacts, slightly better spatial resolution), along with increased dose. The CNR for high contrast materials was best at 85 kVp in the image quality phantom, which was mirrored by the skull phantom results indicating that only 85 kVp and higher could produce diagnostic images for the mandible. From these combined findings, 85 kVp was selected as the optimal value.

For mA optimization, image quality metrics also showed improvements for higher mA for noise and CNR, but improvements for artifacts and spatial resolution, the lower mA values were best. For the skull phantom, all landmarks were visible for 3.2 mA and higher. Selecting 3.2 mA would result in slight improvements in image uniformity compared with the default setting (4 mA) and reduced dose, with slightly more image noise; other parameters such as spatial resolution, geometric distortion, and artifacts would be unchanged, and the loss of CNR would not impact the conspicuity of landmarks. The average dose of the selected settings of 85 kVp and 3.2 mA was 1.2 ± 0.6 mGy, resulting in reduction by approximately 35% compared with the default setting (90 kVp, 4 mA, 1.9 ± 0.3 mGy).

## 4 DISCUSSION

The measurement of the dose with changes in kVp and mA was an important approach to control the applicability of the results as the radiation dose used should comply with ALARA principle (ICRP, 2007). Increases in kVp and mA both yielded an increase in the absorbed dose, with mA showing slightly more impact on the dose as compared with kVp. The Sedentex DI phantom is designed for measuring the absorbed dose in a manner similar to the CTDI metric used with hospital multislice CT scanners (Sedentex Project, 2012). Indeed, the doses measured with the Sedentex DI phantom and methodology yield similar results to the CTDI (Li, Thakur, & Ford, 2017). The absorbed doses reported here are a measure of the energy deposited as a result of the radiation exposure but do not account for the biological effect of the radiation. To measure the biological effects, one must measure the absorbed dose to different tissue types and calculate the effective dose using tissue weighting factors published by the ICRP (2007). To identify the different tissues, an anthropomorphic phantom is required to calculate the effective dose.

Changing the kVp, however, had more impact on image quality than the change in mA. This was consistent with conclusion from a study conducted by Pauwels et al. (2014); the optimization of image quality was done, and both SEDENTEXCT IQ and DI phantoms were used, and the dose was reduced greatly by reducing mA with minor loss of image quality. The quantitative image quality parameters added a value to the optimization by making it more objective as clinical image quality assessment alone is subjective in nature. Higher kVp settings led to improvements in image noise and artifact reduction, but the uniformity across the FOV was reduced. For increased mA, the noise improved, but the uniformity and artifact measurements were best for lower mA. CNR was optimized at 85 kVp for high contrast objects and at 80 kVp for lower contrast objects, with no clear trends related to mA. In this study, the geometrical distortion and spatial resolution measurements did not differ significantly at different settings, suggesting that lower exposure parameters could still obtain adequate image resolution (Abouei et al., 2015; Benavides et al., 2012; Lofthag‐Hansen et al., 2011; Pauwels et al., 2015; Pauwels et al., 2011). However, the interplay between different quantitative parameters and dose is not a straight forward task and requires the selection of a midway exposure setting that satisfies all the needs while maintaining low dose.

In implant dentistry, treatment planning requires assessment of bone volume, quality, orientation, as well as the local anatomy (Bornstein, Scarfe, Vaughn, & Jacobs, 2014). In our study, a natural bone human skull was used, and almost all anatomical landmarks required for implant treatment planning were present. Tracing the inferior alveolar nerve canal is an essential task for implant treatment planning but was not possible to do in the skull we used; the canal was visible only at some cross sections but could not be traced continuously. Miles, Parks, Eckert, and Blanchard (2016) studied the visibility of mandibular canal in CBCT scans, and it was visible in only 56% of the studied subjects, with younger patients (47–56 years) and females showing lower visibility than older patients (65+ years) and males. When the visibility was further studied according to the location, at the premolar site, older males had higher visibility, whereas females had less visibility, compared with molar sites (Miles et al., 2016). Presurgical assessment of the buccal plate thickness is needed to plan the surgical technique in cases of ridge preservation or immediate implant procedures. In a study by Timock et al. (2011), the visibility of the buccal bone thickness and height was evaluated in CBCT. Measurements were verified directly on cadavers, with higher reliability on measurements of buccal bone height than thickness; this finding was consistent with our study. However, we found that the buccal plate thickness of mandibular canine was difficult to identify in our head phantom. Other anatomical landmarks in general had adequate visibility in our study even for the smaller structures such as the antroalveolar intraosseous anastomosis at lateral walls of the sinuses as well as the superior and inferior lingual foramina.

In this study, a 5‐point Likert ranking was used to assess the diagnostic value achieved in the head phantom images over a range of kVp and mA settings. There has been some controversy about the Likert scale recently, with the majority of concerns around the scale used, the meaning of neutrality, how to interpret ordinal data, and what types of statistical tests are appropriate (Keeble et al., 2016; Phelps et al., 2015; Sullivan & Artino, 2013). As recommended by Keeble et al. (2016), we presented the ordinal scale with the numeric and verbal descriptions to the observers, with a clearly understood meaning of 3—*adequate* representing an image of diagnostic value. Statistical testing was not performed; rather the data were binarized, with all imaging tasks receiving scores of 3, 4, or 5 being taken as diagnostic and those ranked as 1 or 2 being unsuitable for clinical use. The results from the maxillary landmarks indicate diagnostic image quality for all kVp settings and for tube current settings as low as 3.2 mA, suggesting a possibility of reducing the dose. However, the anatomical landmarks of mandible required higher exposure settings. Because the CS 9300 does not allow for multiple preset technique factors specific to each arch, 85 kVp and 3.2 mA were selected as the optimal settings for the clinical image quality assessment that works for both arches.

In conclusion, the image quality can be optimized at lower dose by reducing the exposure settings as compared with default settings. This study included a comprehensive method to optimize CBCT image quality using dose measurement, quantitative image quality assessment, and clinical image assessment. The optimization of the images is affected by the dose and should be measured together to obtain adequate diagnostic value of images at lowest possible dose complying with ALARA principle. The optimization should be task specific as different tasks may require different settings to produce the required diagnostic value. The assessment of clinical as well as quantitative image quality is required to ensure that adequate diagnostic value is obtained.

## CONFLICTS OF INTEREST

The authors have stated explicitly that there are no conflicts of interest in connection with this article.
